# Institutional Needs

**Published:** 1913-07-26

**Authors:** 


### Institutional Needs.

"LIFEBELT" COFFEE.

INSTITUTIONAL officers, like other people, are sometimes deterred from taking coffee in the evenings because it keeps them awake. Those among them who suffer in this way will, therefore, be interested in the "pure coffee freed from caffeine" which is prepared by the Lifebelt Coffee Company, Limited, 71 Eastcheap, London, E.C. From the point of view of taste and aroma this coffee is unimpeachable and its flavour delicious; and, in so far as sleeplessness after coffee-drinking is the result of the caffeine element in ordinary coffee, the Lifebelt Company's preparation will do away with the evil complained of without sacrificing the pleasant qualities of the beverage. A penny stamp to the Company's address despatched by any of our readers will bring a sample tin and some interesting leaflets on the subject.

AN IDEAL IN PIPES.

("THE BOON PATENT PIPE." W. G. BOONZAIER, 11 BRAMFIELD ROAD, NEW WANDSWORTH, S.W.)

EARNEST smokers usually eschew patent pipes; there is, they will tell you, hardly a better model to-day than that which Sir Walter used when he astonished the Devon country-folk with his noxious "mouth blasts." Those who have tried the "Boon" must reconsider their opinion with regard to what Artemus Ward would have called the cussedness of patent pipes. The principle of the "Boon" is an entirely novel one, yet it is so simple that one wonders why no inventor hit upon it long ago. It is to all intents an inverted pipe, with the bowl so constructed that no ash can be spilt; thus the pipe may safely be smoked in a high wind or placed in the smoker's pocket. This peculiarity alone should make the "Boon" the ideal pipe for motorists, aviators, travellers, and those engaged in out-door occupations. Add to this the attraction that the pipe smokes smoothly, cleanly, the smoke being cool, and, owing to the effective combustion in the bowl, with a minimum of extractives, and the superiority of the "Boon" over other types will easily be acknowledged by those who have tried it. In our opinion it is the most hygienic pipe on the market, and the only patent with which the habitual smoker, used to the ease of the ordinary briar, will be satisfied. Sample pipes may be obtained direct from the patentee at the address given above.

